# The impact of plyometric and small-sided games training on physical performance in adolescent female handball players

**DOI:** 10.3389/fspor.2026.1812244

**Published:** 2026-04-07

**Authors:** Attila Beszkid, Zsófia Mészáros, Katalin Kovács

**Affiliations:** 1 Faculty of Education and Psychology, Doctoral School of Education, Eötvös Loránd University, Budapest, Hungary; 2 Faculty of Education and Psychology, Institute of Health Promotion and Sport Sciences, Eötvös Loránd University, Budapest, Hungary

**Keywords:** adolescence, performance testing, physical performance, plyometric training, small-sided games, training intervention, youth handball

## Abstract

**Introduction:**

Plyometric training (PLYO) and small-sided games (SSG) are widely used to improve explosive power and agility in youth team sports, yet field-test evidence in pubertal athletes is limited. The aim of this study was to compare the effects of a six-week PLYO and SSG training program on jumping, sprinting, and change-of-direction (5-10-5 agility test) in adolescent female handball players.

**Methods:**

A non-randomized, parallel-group intervention was conducted involving three intact U16 female handball teams from the Budapest area. Teams were assigned to plyometric training (PLYO; *n* = 12), small-sided games (SSG; *n* = 12), or standard training (control; CTRL; *n* = 10). The six-week intervention was integrated into regular practice. The PLYO and SSG groups performed their assigned training tasks twice per week, while the control group continued usual training. Performance was assessed with vertical jump (VJ), standing long jump (SLJ), 20 m sprint, and 5–10–5 agility test; the best of three trials was analyzed. Within-group changes were evaluated with paired t-tests and effect sizes (Cohen's d), while between-group differences were examined using one-way ANOVA.

**Results:**

Comparing pre-test results, a significant difference was observed in VJ between PLYO and SSG (*p* = 0.030). No other baseline differences were detected. Over 6 weeks, PLYO improved across all tests, with significant changes in VJ (*p* = 0.001), SLJ (*p* = 0.002), 20 m sprint (*p* < 0.001), and 5–10–5 (*p* = 0.002). SSG showed no meaningful changes in VJ (*p* = 0.958) or SLJ (*p* = 0.345), but significant improvements in 20 m sprint (*p* = 0.038) and 5–10–5 (*p* < 0.001). CTRL significantly improved sprint (*p* = 0.015) and 5–10–5 (*p* < 0.001) with minimal changes in jump outcomes. Post-test between-group differences were not statistically significant.

**Discussion:**

Taken together, these findings suggest that both PLYO and SSG may be integrated into youth handball training, with potential modality-specific benefits. Larger randomized studies are required to clarify their comparative effects and optimal programming.

## Introduction

Handball was a dynamic and high-intensity sport that demanded both high-level physical and cognitive abilities from players. It was understood that its performance required a certain level of physiological capacity, particularly with regard to the ability to generate and repeat the kind of explosive strength that are necessary for sprinting, jumping and change of direction.

In recent years, the sport's pace has accelerated significantly. Karcher and Buchheit (1) highlighted that handball players engaged in high-intensity actions approximately every 23 s on average.

Additionally, recent rule modifications (2) have further amplified the physical demands on athletes. This ability was considered to be important not only for adult players, but also for adolescent players.

The origins of plyometric training dated back to the 1950s, when Russian coach Yuri Verkhoshansky first introduced depth jumps into the training programs of his athletes. The method first became known to the public as the “Shock Method”. Plyometric training was rooted in the stretch-shortening cycle (SSC), a neuromuscular mechanism that played a fundamental role in the development of explosive power and muscular strength. The SSC enabled muscles to store elastic energy during the eccentric (stretching) phase and release it efficiently during the concentric (shortening) phase, thereby enhancing performance in key movements such as jumping, sprinting, and rapid direction changes (3).

Plyometric training was essential in developing reactive strength, focusing on neuromuscular adaptations rather than inducing significant muscle hypertrophy. Instead of primarily targeting muscle mass, this training method enhanced the stiffness of the muscle-tendon complex and refined the responsiveness of the central nervous system (3, 4). Current research on plyometric training recommended incorporating two targeted sessions per week over the course of 1–2 microcycles (typically 4 to 8 weeks) to achieve optimal performance improvements (3). Ruppert et al. (5) conducted a study on female youth handball players, exploring the impact of plyometric exercises on their performance. The participants were divided into two groups: one that had plyometric exercises once per week and another that performed twice per week. Based on their results, Patel (3) was reported, that performing plyometric exercises twice per week is more effective.

In team sports, a skilled player could select the optimal option from a range of possibilities in a remarkably brief period of time. Decision making was one of the most important performance factors in team sports today. Decisions were influenced by skill, knowledge, prior experience and of course perception. SSG games could simulate match situations and provide the right stimulus for the development of physical ability (6). While usually approached in the literature from a tactical—technical point of view, some studies analysed the effect of SSG on endurance, agility or explosiveness (7).

SSG games were often used by physical education teachers and coaches in their training sessions. In these games, the number of players and field sizes were reduced, and the rules could be modified (7–10).

Previous studies of plyometric and SSG training that uses field tests in pubertal children were limited (11–22).

Direct comparisons between plyometric training and SSG-based training programs using field-based performance tests in pubertal female athletes remained scarce. Furthermore, adolescent female handball players represented a population in which neuromuscular development, training adaptation, and sport-specific demands might differ from those reported in male cohorts. Previous studies have shown that plyometric training can significantly improve jumping ability, sprint performance, and change-of-direction ability in youth female handball players (17, 22). This observation may also suggest that both training approaches could contribute to the development of complementary physical qualities in youth handball players. Consequently, integrating or periodising plyometric exercises alongside small-sided games within the weekly training structure might provide a balanced stimulus for improving multiple components of physical performance.

In the aforementioned plyometric articles, an improvement of 13%–15% was observed in jumping performance, while in the SSG articles, the average improvement was 7%. For sprint and agility tests, the plyometric training resulted in a 4%–5% improvement, while the methods applying SSG games showed a 1%–2% improvement. The highest improvement, 12%–13%, was observed in the group that regularly performed numerous jumps in their sport (basketball) and supplemented this with SSG.

In two articles (16, 19) where plyometric training was combined with sprint training, higher improvements were achieved — a 30% increase in jumping and an 8% increase in sprint performance.

In a systematic review by Hammami et al. (23), which included 15 studies, the effects of SSG on physical performance were examined. According to their research, the impact of SSG on field tests was moderate or medium for jumping and sprint tests, while it was more effective in sport-specific agility, endurance, or sport-specific technical tasks compared to general tasks.

In response to this gap, the present study examined and compared the effects of plyometric training and small-sided games on physical performance in adolescent female handball players. The study specifically evaluated changes in vertical jump, standing long jump, sprint performance, and change-of-direction ability following a six-week training intervention.

It was hypothesised that plyometric training would be associated with greater improvements in jumping performance, while small-sided games would be associated with greater improvements in sprint and change-of-direction performance due to the movement patterns embedded in each training modality. By comparing these approaches within a field-based training context, the study aimed to contribute to a better understanding of how different training methods influenced physical development in youth female handball players.

## Materials and methods

### Study design

The study followed a quantitative, non-randomized, parallel-group pre–post intervention design conducted under applied field-based training conditions. Such designs are commonly used in sport performance research to evaluate training-related adaptations in athletes within ecological sport environments (24). The purpose of the investigation was to compare the effects of two training approaches—plyometric training (PLYO) and small-sided games (SSG)—with a control condition involving regular handball training.

### Participants

Three female handball teams from the Budapest metropolitan area participated in the study, comprising a total of 45 players. Due to injuries, illnesses, and other absences, 34 players completed the post-assessment phase. All participants held valid competition licenses and had a minimum of two years of prior handball training experience, with a regular training frequency of at least three sessions per week (Table 1).

**Table 1:** Participant groups’ data.

Group	Participants (*n* = 34)	Age (years)	Prior experience (years)	Weekly training (hours)	Weekly strength training (hours)
CTRL	10	14.17	3	6	0
PLYO	12	14.66	3.5	7.5	1.5
SSG	12	14.36	3.6	7	1.5

The teams competed in youth handball championships, ensuring a comparable level of skill and experience among participants. We conducted a non-randomized, parallel-group study with three intact U16 female handball teams. One team followed a plyometric training program (PLYO), one performed small-sided games (SSG), and one continued usual training (control, CTRL). The CTRL group did not participate in any additional training beyond their regular handball practice sessions.

Participants' age was determined as calendar age expressed in the decimal numeral system (decimal years), following the calculation procedure described by Weiner and Lourie (25). Based on the available data, biological maturity could not be directly assessed. However, considering the median age at menarche, it was assumed that the girls' biological development was already approaching adult maturity.

### Training program

A six-week intervention was conducted, with training sessions integrated into regular practice sessions. All training sessions were conducted after an appropriate warm-up routine to ensure participants were in an optimal physiological state.

The plyometric training group (PLYO) incorporated vertical, horizontal, and depth jumps, into strength training sessions on Mondays, with an additional session either on Wednesdays or Thursdays, depending on match schedules. Training emphasized a progressive overload approach, increasing intensity over time.

The SSG training group performed their prescribed training twice per week, on Tuesdays and Thursdays, using modified handball drills and preparatory games.

The CTRL group continued with their standard training regimen, which included technical and tactical exercises but no additional plyometric or SSG drills.

The progression of each training program was monitored and supervised by team coaches to ensure consistency and compliance.

### Plyometric training protocol

The plyometric training program was designed to develop reactive strength, explosiveness, and neuromuscular efficiency. Exercises were selected based on SSC principles, ensuring a combination of fast SSC (ground contact <250 ms) and slow SSC (ground contact >250 ms) movements. Recommendations for plyometric training in children were followed based on the systematic review by Johnson et al. (26).

According to prior recommendations, plyometric load was progressed weekly by increasing the number of jumps. In our program (Supplementary Table S1), we increased weekly volume solely via ground contacts, progressing from 50 in Week 1 to 105 by Week 6.

Exercise Selection:

The plyometric program included the following categories of exercises:

1. Vertical Jumps (e.g., squat jumps, tuck jumps)
2. Horizontal Jumps (e.g., broad jumps, bounding drills)
3. Depth Jumps (e.g., drop jumps from varied heights)
4. Lateral Jumps (e.g., skater jumps, side-to-side bounds)

The progressive overload approach involved:

- Weeks 1–4: Gradual increase in repetitions or intensity
- Weeks 5–6: Reduction in ground contact frequency, with an emphasis on eccentric workload

### Small-sided games (SSG) training protocol

SSG training consisted of modified handball drills and preparatory games, ensuring a dynamic and game-specific conditioning approach.

- Team size: Maximum 4v4 format was utilized
- Session structure: The structure was adapted based on attendance, allowing for 2-team or 4-team rotations (e.g., 3v4 or 4v3 formats).
- Game rules: Rules were adjusted to enhance decision-making, agility, and tactical awareness, and all games were played without a goalkeeper.

SSG sessions were supervised by team coaches and emphasized high-intensity, game-relevant movements while fostering tactical adaptability. No additional external load was introduced beyond regular handball training during the intervention period. Detailed drill configurations are provided in Supplementary Table S2.

### Assessments

Baseline (Pre-Intervention) Assessments were conducted on October 29–30, 2024, during the competitive season (in-season) and coincided with the autumn school break. This timing ensured that participants were well-rested before testing. Athletes refrained from competition and high-intensity training prior to testing. A standardized warm-up was performed, including mobility drills, moderate-intensity running, and plyometric activation exercises.

Post-Intervention Assessments were conducted on December 16–17, 2024, also during the competitive season (in-season), after the completion of scheduled matches and immediately before the winter break. Testing conditions were identical to those used during the baseline assessments.

Each participant was provided with three test attempts, with the best result recorded for statistical analysis.

Sprint and agility times were recorded using manual stopwatches. Timing was performed simultaneously by two independent evaluators—the team coach and the principal investigator—using separate stopwatches. The recorded time for each trial was calculated as the average of the two measurements to reduce potential human timing error. Jump performances were measured by the principal investigator. Standing long jump distance was recorded using a measuring tape, while vertical jump height was assessed using a vertical reach measuring device (Vertec-type apparatus)**.** All tests were conducted indoors under standardized environmental conditions.

### Performance tests

Performance was evaluated through the following tests

1. Vertical Jump Test (VJ)—Measures explosive lower-body power (27).
2. Standing Long Jump Test (SLJ)—Evaluates horizontal power output and dynamic leg strength (28).
3. 20-Meter Sprint Test—Assesses acceleration and sprinting capability (29).
4. Change of Direction Test (5-10-5 agility test)—Evaluates agility and reactive movement efficiency (30).

All tests were performed in an indoor sports hall under controlled conditions to ensure consistency across trials.

### Data collection and statistical analysis

All data were recorded on Microsoft Excel and R software was used for statistical analysis. Pre- and post-assessment data were averaged, and standard deviations (SD) were calculated. Percentage changes in performance were computed relative to pre-test values, which were arbitrarily set at 100%.

Prior to inferential statistical analysis, normality of distribution was assessed using the Shapiro–Wilk test, and homogeneity of variances was evaluated using Levene's test.Within-group changes between pre- and post-intervention measurements were analyzed using paired-sample t-tests. Between-group differences were examined using one-way analysis of variance (ANOVA).

Cohen's d effect size was calculated to determine the magnitude of training effects, using the classification by Cohen (31).

- small effect: ≥0.2
- moderate effect: ≥0.5
- large effect: ≥0.8

Statistical significance was set at *p* < 0.05.

## Results

### Pre- and post-assessment results

The following section presents the results of the six-week intervention, comparing the pre- and post-assessment performance of the PLYO group, SSG group, and CTRL group across key physical performance metrics. These include vertical jump (VJ), standing long jump (SLJ), 20-meter sprint, and the 5-10-5 agility test.

The pre- and post-assessments were carried out at the end of October and in the middle of December. Statistical procedures were applied to the results of the field tests to identify differences between the groups.

In the pre-test results (Table 2), a significant difference was found in the vertical jump (*p* = 0.031), and the Tukey's HSD *post hoc* test revealed a significant difference between the PLYO and SSG groups. No other significant differences were observed in the baseline results for any other metrics.

**Table 2:** Statistical results of preliminary performance between groups.

Pre-test group comparison							
	Groups			Statistical analyses (*p*-value)			
Performance tests	PLYO	SSG	CTRL	ANOVA	Tukey's PLYO:SSG	Tukey's PLYO: CTRL	Tukey's SSG:CTRL
VJ (cm)	37.29 ± 4.6	42.63 ± 3.9	39.50 ± 5.6	0.031*	0.030*	0.510	0.720
SLJ (cm)	173.00 ± 19.31	184.17 ± 14.06	186.20 ± 19.47	0.174	0.310	0.200	0.960
20 m sprint (s)	3.60 ± 0.18	3.46 ± 0.17	3.60 ± 0.18	0.111	0.170	0.990	0.170
5-10-5 (s)	5.53 ± 0.27	5.50 ± 0.30	5.50 ± 0.17	0.692	0.990	0.770	0.690

* After statistical analyses, a significant difference was observed between the PLYO and SSG groups (*p* < 0.05).

The post-tests assessments were conducted at the same location (sports hall) and on the same day of the week and the same time of day as the pre-tests. Post-intervention between-group comparisons (Table 3) revealed no statistically significant differences between groups in vertical jump (*p* = 0.209), standing long jump (*p* = 0.724), 20 m sprint (*p* = 0.340), or the 5–10–5 change-of-direction test (*p* = 0.932).

**Table 3:** Statistical results of post-intervention performance between groups.

Post-test group comparison							
	Groups			Statistical analyses (*p*-value)			
Performance tests	PLYO	SSG	CTRL	ANOVA	Tukey's PLYO:SSG	Tukey's PLYO:CTRL	Tukey's SSG:CTRL
VJ (cm)	43.25 ± 4.8	42.58 ± 4.46	39.50 ± 5.17	0.209	0.940	0.200	0.340
SLJ (cm)	186.00 ± 15.89	182.25 ± 16.77	187.70 ± 17.86	0.724	0.830	0.980	0.720
20 m sprint (s)	3.47 ± 0.19	3.38 ± 0.13	3.50 ± 0.18	0.340	0.460	0.990	0.390
5-10-5 (s)	5.33 ± 0.23	5.31 ± 0.25	5.30 ± 0.2	0.932	0.990	0.920	0.970

No statistically significant differences were observed between the groups (*p* > 0.05).

### Vertical jump (VJ)

The PLYO group exhibited a significant improvement in VJ, increasing by 16% (*d* = 1.26, *p* = 0.001), confirming the effectiveness of SSC-based plyometric training in enhancing explosive strength. In contrast, the SSG group (*p* = 0.958, *d* = 0.011) and the CTRL group (*p* = 0.212, *d* = 0.002) did not show meaningful changes, aligning with expectations given the nature of their respective training interventions (Table 4).

**Table 4:** Results of the post-field tests and their within-group statistical comparison.

	PLYO group					SSG group					CTRL group				
Performance tests	Before (Mean ± SD)	After (Mean ± SD)	Change (%)	Cohen's d (Effect Size)	*T*-test (*p*-value)	Before (Mean ± SD)	After (Mean ± SD)	Change (%)	Cohen's d (Effect Size)	*T*-test (*p*-value)	Before (Mean ± SD)	After (Mean ± SD)	Change (%)	Cohen's d (Effect Size)	*T*-test (*p*-value)
VJ (cm)	37.29 ± 4.6	43.25 ± 4.8	16	1.26	0.001*	42.63 ± 3.9	42.58 ± 4.46	−0.1	0.011	0.958	39.50 ± 5.6	39.50 ± 5.17	0.1	0.002	0.212
SLJ (cm)	173.00 ± 19.31	186.42 ± 15.89	7.8	0.75	0.002*	184.17 ± 14.06	182.25 ± 16.77	−1.4	0.12	0.345	186.20 ± 19.47	187.70 ± 17.86	0.8	0.008	0.663
20 m sprint (s)	3.60 ± 0.18	3.47 ± 0.19	3.8	0.7	0.001*	3.46 ± 0.17	3.38 ± 0.13	2.36	0.52	0.038*	3.60 ± 0.18	3.50 ± 0.18	3.6	0.72	0.015*
5-10-5 COD (s)	5.53 ± 0.27	5.33 ± 0.23	3.8	0.79	0.002*	5.50 ± 0.30	5.31 ± 0.25	4.41	0.83	0.001*	5.50 ± 0.17	5.30 ± 0.2	3.1	0.1	0.001*

* Statistically significant within-group difference (*p* < 0.05).

### Standing long jump (SLJ)

The PLYO group demonstrated a 7.8% increase in SLJ performance (*d* = 0.75, *p* = 0.002), reinforcing the benefits of plyometric training on horizontal power development (Table 2). The SSG group (*p* = 0.345, *d* = 0.12) and the CTRL group (*p* = 0.663, *d* = 0.008) exhibited no significant changes (Table 4).

### 20-meter sprint

Sprint performance improved across all groups. The PLYO group displayed the highest gains (3.8%, *p* < 0.001, *d* = 0.70), followed by the SSG group (*p* = 0.038, *d* = 0.52) and the CTRL group (*p* = 0.015, *d* = 0.72) (Table 4).

### 5-10-5 agility test

Agility improvements were observed in all groups, though the SSG group showed the largest effect (*p* < 0.001, *d* = 0.83), closely followed by the PLYO group (*p* = 0.002, *d* = 0.79). The CTRL group exhibited only minimal improvements (*p* < 0.001, *d* = 0.10) (Table 4).

## Discussion

In the 6-week intervention program, three female handball teams participated, with two groups following an SSG-based and a plyometric training program, respectively. These programs were integrated into regular training sessions twice a week as training blocks. The CTRL group did not receive any additional training. To evaluate the impact of these interventions, field test results (VJ, SLJ, 20 m sprint, 5-10-5 agility test) were compared among the three groups, following similar methodologies used in prior research on youth handball performance (15, 16).

Regarding changes in the VJ test, the PLYO group exhibits a notable within-group improvement (16%), with a statistically significant increase and a strong effect size. Importantly, the PLYO group starts from the lowest baseline level in VJ performance, which **is** a factor to consider when interpreting the larger relative improvement observed. The SSG and CTRL groups show no meaningful changes, as their performance remains comparable to their baseline values.

However, it is crucial to note that no statistically significant between-group differences are observed at post-test. Therefore, although within-group improvements are evident, these findings do not support definitive conclusions regarding the superiority of one training method over the other.

To assess developmental progress several studies on adolescent plyometric training use field tests (13–17, 22, 32, 37). Hammami et al. (16) report a ∼30% improvement in a similar age group of male handball players after an 8-week intervention, while their control group shows only minimal change (+0.1%), which is consistent with the pattern observed in the present results. The observed 16% vs. 30% difference in vertical jump improvements may be attributed to differences in intervention duration (6 weeks vs. 8 weeks) and gender differences. Previous studies suggest that male athletes may exhibit greater neuromuscular adaptations in response to plyometric training due to higher baseline strength levels and testosterone-driven hypertrophy (32, 33).

For the SLJ test, the plyometric group demonstrates an 8% improvement, which aligns with existing research. Studies indicate that twice-weekly plyometric training for six weeks can significantly enhance both vertical and horizontal jump performance in adolescent athletes (15–17, 22).

This study and the existing literature indicate that plyometric training programs produce improvements in adolescent athletes' performance metrics. Specifically, jump tests (e.g., standing vertical jumps) demonstrate enhancements ranging from 13% to 15%. Similarly, 20-meter sprint and the 5-10-5 agility tests reveal average improvements of approximately 4.5% following such training interventions. Notably, these performance gains are more pronounced among athletes who incorporate plyometric exercises alongside their regular sport-specific training. This suggests that integrating plyometric training with standard athletic routines may contribute to improvements in both sprinting and jumping capabilities.

In contrast to previous research, the SSG group shows no improvement in jump tests (VJ: −0.1%; SLJ: −1.4%). This contradicts findings from Karahan (18), Arslan et al. (21), and Jurisic et al. (20), which reported 3%–10% improvements in jump performance following SSG-based interventions.

Discrepancies in SSG jump results may be attributed to several factors. (1) Training specificity: jumping performance improvements are largely influenced by maximal force production and neuromuscular adaptations, which may not be effectively stimulated in SSG-based training compared to dedicated plyometric training (17). (2) Fatigue accumulation: unlike controlled plyometric training, SSG sessions involve continuous high-intensity movement, potentially leading to higher fatigue levels, which masks neuromuscular adaptations (34). (3) Variability in game format: differences in team size, rule modifications, and work-to-rest ratios in SSG studies affect their efficacy in developing power-related attributes (9). Given that jump tests primarily depend on lower limb maximal strength, and neither SSG nor the CTRL group includes dedicated strength training, these findings are expected. However, a slight improvement in the SSG group is anticipated based on prior literature.

All groups show improvements in 20 m sprint performance. The PLYO group demonstrates the greatest improvement (4%), with statistical significance (*p* < 0.001, *d* = 0.70). However, Cohen's d effect size indicated that the CTRL group experienced the largest relative change (d = 0.72). This may be due to their smaller sample size (*n* = 10), where even a minor improvement (0.1 s reduction) had a considerable statistical impact. Previous studies have also shown moderate-to-high sprint performance improvements following plyometric training interventions, supporting our findings (12, 17).

As expected, the 5-10-5 agility test showed improvements across all. The SSG group demonstrates the highest change (4.5%) with a strong effect size (*d* = 0.83, *p* < 0.001). This result is consistent with previous findings, as small-sided games involve frequent decelerations, changes of direction, and accelerations, closely simulating the demands of the 5-10-5 test (7). Interestingly, even the CTRL group shows improvement in their agility performance (3.1%). This suggests that familiarity with movement patterns may play a greater role in complex movement tasks than in sprinting or jumping performance. Chaouachi et al. (35) report similar results in 14.2-year-old soccer players, where the CTRL group showed minimal gains in CMJ (Countermovement Jump) but exhibited greater percentage improvements in agility tests.

## Key findings and practical implications

1. Plyometric training was associated with greater within-group improvements in VJ, SLJ, and 20 m sprint, reinforcing its role in enhancing explosive power and neuromuscular efficiency.
2. SSG training improves agility and sprint performance but shows limited effects on jumping ability, suggesting that it is more suited for sport-specific reactive movement development rather than maximal power output.
3. Overall, the findings suggest a modality-specific pattern of adaptations, with plyometric training being more strongly associated with jump-specific adaptations (alongside improvements in sprint and agility), while SSG tended to favor agility and sprint outcomes with little to no improvement in jumping performance.
4. CTRL group improvements in sprint and agility may be explained by familiarity with testing procedures and natural neuromuscular adaptation over time, although gains are less pronounced than in the experimental groups. These results should also be interpreted cautiously, as the CTRL group has the smallest sample size, which may influence the magnitude of observed effect sizes and relative changes.

## Conclusion

The study compares two widely used training methods in youth team sports. While the effects of plyometric training are well documented, evidence on the impact of small-sided games on physical performance—especially in adolescent female handball using field-based pre–post designs—remains limited.

Across six weeks, a modality-specific pattern of adaptations is observed. Plyometric training is associated with gains in jumping alongside improvements in sprint and 5-10-5 agility test, whereas SSG tends to favor agility and sprint outcomes with little change in jumping. These patterns are consistent with the movement demands embedded in each modality (e.g., rapid stretch–shortening and explosive take-offs in PLYO; frequent accelerations, decelerations, and directional transitions in SSG) and align with literature suggesting heightened neuromuscular responsiveness in younger athletes (15, 17).

Limitations of this study include the small sample size and the fact that the teams (groups) are trained under different circumstances including training environment, coaching staff and training content. Furthermore, the accuracy of the field tests is limited compared with laboratory-based measurement methods. These factors influence the homogeneity and generalisability of the findings. It is also noted that post-test between-group differences are not significant, and the design is non-randomized, therefore, neither method is declared superior to the other based on the current results.

Several methodological factors require a critical interpretation of these findings. First, the statistical power is constrained by the small sample size, which reduces the likelihood of detecting significant between-group differences despite the observed within-group improvements. Second, biological maturation is not formally assessed; in U16 female athletes, variations in maturation status significantly influence neuromuscular adaptations, potentially confounding the intervention effects. Furthermore, the use of intact teams introduces potential coaching effects, as differences in training culture or motivational climates between teams may affect performance outcomes.

An additional limitation is that handball playing positions are not analysed separately (e.g., backcourt, wing, pivot, goalkeeper), which may have obscured position-specific responses to PLYO vs. SSG given the differing physical demands across roles.

From a practical perspective, coaches working with adolescent female handball players may consider implementing plyometric training when the primary goal is to improve jumping performance. Small-sided games may be particularly useful for developing sprint and change-of-direction abilities within a more game-specific training context. Combining or periodising both training approaches within the weekly training structure may help address complementary physical demands.Future research may benefit from the use of standardised measurement tools (e.g., photocells, force plates) and larger and more diverse samples including different backgrounds, genders, intervention durations and age groups. Further studies may also examine positional differences in handball to determine whether the effects of plyometric training and small-sided games vary according to position-specific performance profiles and physical demands.

## Data Availability

The original contributions presented in the study are included in the article/Supplementary Material, further inquiries can be directed to the corresponding author/s.
