# Supplementary material for: The impact of plyometric and small-sided games training on physical performance in adolescent female handball players
Source: Front Sports Act Living. 2026 Apr 7;8:1812244. doi: 10.3389/fspor.2026.1812244 (PMC13095714; doi:10.3389/fspor.2026.1812244)
Supplement: Supplementary file 1 [file Table1.docx]

**Supplementary Table 1.** Detailed plyometric training program outlining the weekly microcycles, the number of sets and repetitions per session, rest intervals, and total ground contacts per week

| **Microcycles (Weeks)** | **Sets / Reps / Set Rest / Rest Between Exercises (sec)** | **Ground Contacts per Week** |
| --- | --- | --- |
| 1 | 5 selectable exercises: 2x5 / 40 sec / 30 sec | 50 |
| 2 | 5 selectable exercises: 2x6 / 40 sec / 30 sec | 60 |
| 3 | 5 selectable exercises: 2x7 / 40 sec / 45 sec | 70 |
| 4 | 5 selectable exercises: 2x8 / 40 sec / 45 sec | 80 |
| 5 | 5 selectable exercises: 3x6 / 50 sec / 60 sec | 90 |
| 6 | 5 selectable exercises: 3x7 / 50 sec / 60 sec | 105 |
