# Supplementary material for: The impact of plyometric and small-sided games training on physical performance in adolescent female handball players
Source: Front Sports Act Living. 2026 Apr 7;8:1812244. doi: 10.3389/fspor.2026.1812244 (PMC13095714; doi:10.3389/fspor.2026.1812244)
Supplement: Supplementary file 2 [file Table2.docx]

**Supplementary Table 2.** Overview of the small-sided games training program, detailing the weekly microcycles, the number of games per session, work-to-rest ratios, and total accumulated work duration.

| **Microcycles (Weeks)** | **Number of Games** | **Work / Reps / Set Rest / Rest Between Games (sec)** | **Total Work Duration (min)** |
| --- | --- | --- | --- |
| 1 | 2 games | 90x3 / 45 / 60 sec | 9 |
| 2 | 2 games | 100x3 / 45 / 60 sec | 10 |
| 3 | 3 games | 70x3 / 35 / 60 sec | 10.5 |
| 4 | 3 games | 80x3 / 40 / 60 sec | 12 |
| 5 | 3 games | 85x3 / 40 / 60 sec | 12.75 |
| 6 | 3 games | 90x3 / 45 / 60 sec | 13.5 |
